# Acid ceramidase controls apoptosis and increases autophagy in human melanoma cells treated with doxorubicin

**DOI:** 10.1038/s41598-021-90219-1

**Published:** 2021-05-27

**Authors:** Michele Lai, Rachele Amato, Veronica La Rocca, Mesut Bilgin, Giulia Freer, Piergiorgio Spezia, Paola Quaranta, Daniele Piomelli, Mauro Pistello

**Affiliations:** 1 Retrovirus Centre, Department of Translational Medicine and New Technologies in Medicine and Surgery, University of Pisa, Pisa, Italy; 2 Institute of Life Science, Scuola Sant’Anna Pisa, Pisa, Italy; 3 Cell Death and Metabolism Unit, Center for Autophagy, Recycling and Disease, Danish Cancer Society Research Center, Copenhagen, Denmark; 4 Anatomy and Neurobiology, University of California, Irvine, CA USA; 5 Virology Unit, Pisa University Hospital, Pisa, Italy

**Keywords:** Autophagy, Cell death, Cancer therapeutic resistance, Chemotherapy, Cancer

## Abstract

Acid ceramidase (AC) is a lysosomal hydrolase encoded by the *ASAH1* gene, which cleaves ceramides into sphingosine and fatty acid. AC is expressed at high levels in most human melanoma cell lines and may confer resistance against chemotherapeutic agents. One such agent, doxorubicin, was shown to increase ceramide levels in melanoma cells. Ceramides contribute to the regulation of autophagy and apoptosis. Here we investigated the impact of AC ablation via CRISPR-Cas9 gene editing on the response of A375 melanoma cells to doxorubicin. We found that doxorubicin activates the autophagic response in wild-type A375 cells, which effectively resist apoptotic cell death. In striking contrast, doxorubicin fails to stimulate autophagy in A375 AC-null cells, which rapidly undergo apoptosis when exposed to the drug. The present work highlights changes that affect melanoma cells during incubation with doxorubicin, in A375 melanoma cells lacking AC. We found that the remarkable reduction in recovery rate after doxorubicin treatment is strictly associated with the impairment of autophagy, that forces the AC-inhibited cells into apoptotic path.

## Introduction

Sphingolipids are bioactive lipids that play important structural and signaling roles in eukaryotic cells^1, 2^. Ceramides are considered the hub of sphingolipid metabolism and have been implicated in the regulation of multiple cellular processes, including growth inhibition, apoptosis, senescence and autophagy^3–6^. Most notably, intracellular accumulation of long-chain ceramides activates a pro-apoptotic cellular environment^7, 8^. Contrary to ceramides, sphingosine-1-phosphate (S1P) exerts pro-survival effects that favor cell growth, cell motility, migration, and angiogenesis^9^. The balance between the cellular levels of these two sphingolipids is thought to play important roles in the control of cell fate^10, 11^.

Humans express five ceramidases—enzymes that convert ceramide into sphingosine (Shp) and fatty acid—which are classified based on their cellular localization, primary structure and pH optimum (alkaline, neutral or acid). Neutral ceramidases are involved in the digestion of dietary sphingolipids and are localized to the intestinal tract^12^. Alkaline ceramidases are involved in the regulation of cell differentiation, DNA damage-induced apoptosis and cell cycle progression^13–15^. Acid ceramidase (AC, encoded in humans by the *ASAH-1* gene) is a 50 kDa enzyme that belongs to the N-terminal nucleophile (Ntn) superfamily of hydrolases^16^. It is synthesized as an inactive proenzyme that matures through autocleavage of an internal peptide bond, which generates a catalytically competent heterodimer comprising a 13 kDa α-subunit and a 30 kDa β-subunit. AC is the only ceramidase that requires an ancillary protein, saposin-D, to achieve optimal activity^16^. Saposins are lysosomal proteins that enable the presentation of the ceramide substrate to the substrate-binding site of AC.

Mutations in the *ASAH-1* gene give rise to a rare group of genetic disorders that include Farber disease and spinal muscular atrophy with progressive myoclonic epilepsy (SMA-PME)^17, 18^. Elevation in AC expression have been documented in several types of cancers including melanoma, prostate cancer, acute myeloid leukemia and glioblastoma^4, 19, 20^. AC expression has been linked to tumor progression and resistance to chemotherapy and radiotherapy^19, 21, 22^. Indeed, ceramide accumulation is considered one of the mechanisms through the anthracycline chemotherapeutic agent, doxorubicin^23^, exerts its pro-apoptotic effects. Doxorubicin affects sphingolipid metabolism^24^ and heightens the intracellular levels of sphingosine (Shp)^23^, the precursor for S1P, and long chain ceramides such as Cer d18:1–16:0^23–25^. The latter compounds are involved in two distinct, but mechanistically linked, processes: autophagy and apoptosis^26–28^. Such process share common upstream trigger-signals that can lead to develop a cell phenotype in which both are expressed^26^. In many other cases, however, cells switch between autophagy and apoptosis in a mutually exclusive manner^26^.

AC expression is higher in normal human melanocytes and proliferative melanoma cell lines, compared with other skin cells and non-melanoma cancer cells^4^. High AC expression was also observed in biopsies from human subjects with Stage II melanomas^4, 6^.

Recent advances in cancer targeted therapy associates PEGylated C16-ceramides and doxorubicin to enhance the therapeutic response to doxorubicin^29^. Considering that AC plays a major role in melanoma chemoresistance^4, 5^ and considering that ceramides increase doxorubicin cytotoxicity, we hypothesize that AC inhibition might restore melanoma sensitivity to doxorubicin. Indeed, in the absence of AC, melanoma cells wouldn’t be able to counteract the doxorubicin-induced ceramide accumulation and the consequent activation of apoptotic signals.

As resistance to chemotherapeutic agents and relapse of advanced melanoma remains an urgent medical need^30^, in the present study we asked whether AC deletion via CRISPR-Cas9 gene editing might affect the response of human A375 melanoma cells to doxorubicin. Our results show that genetic AC removal impairs autophagy and forces cells treated with doxorubicin to undergo apoptosis.

## Results

### AC-null cells are susceptible to doxorubicin

**Figure 1:**
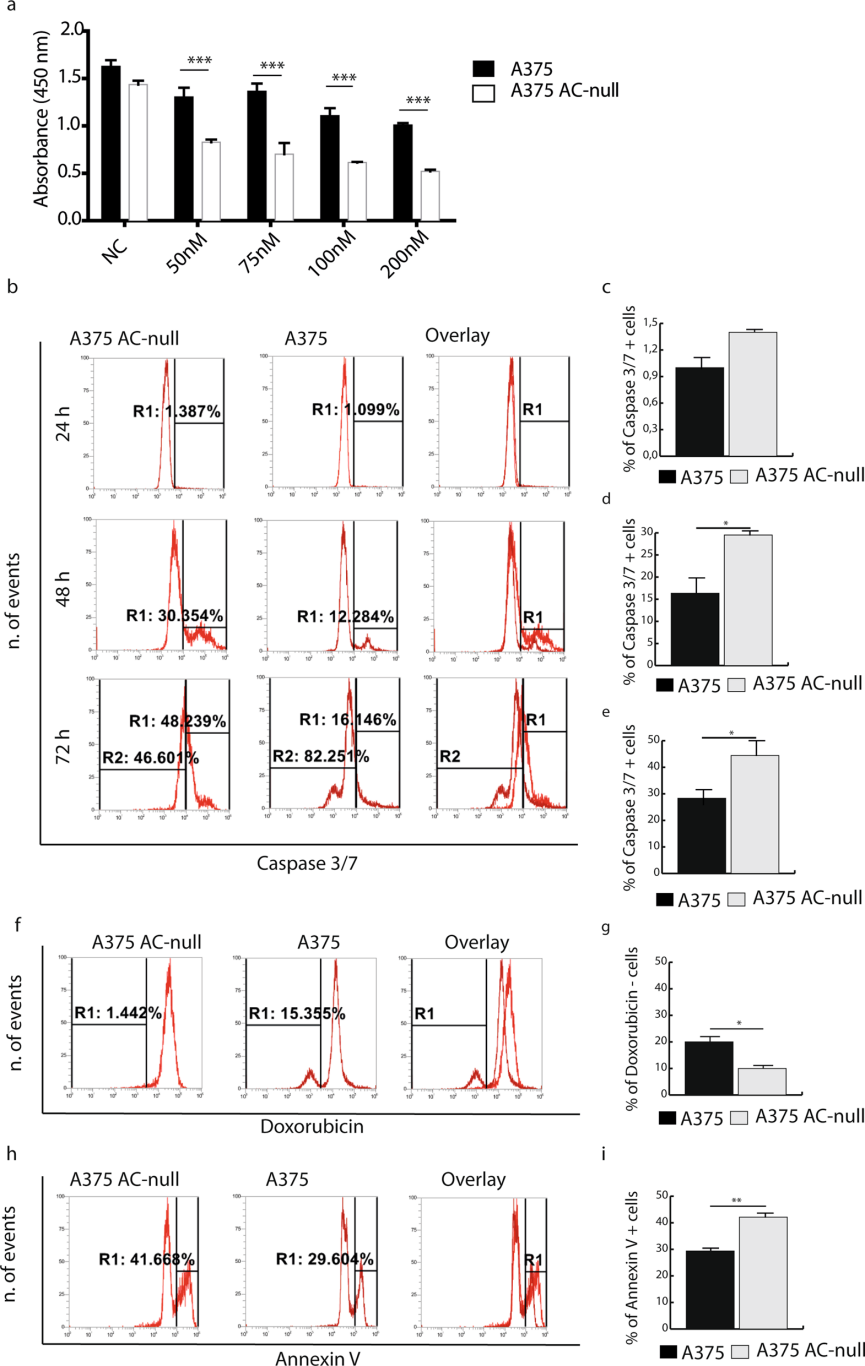
A375 melanoma cells are more resistant to doxorubicin compared to A375 AC-null cells. (**a**) Vitality assay performed on A375 and A375 AC-null cells 48 h after doxorubicin treatments, data are expressed as mean ± SD, One-way ANOVA followed by Tukey’s test were used for statistical analyses, experiments were performed in three independent experiments with three technical replicates. (**b**) Caspase 3/7 assay performed 24 h after 50 nM doxorubicin treatment. (**c**) Statistical analysis of Caspase 3/7 assay performed 24 h after 50 nM doxorubicin treatment. (**d**) Statistical analysis of Caspase 3/7 assay performed 48 h after 50 nM doxorubicin treatment. (**e**) Statistical analysis of Caspase 3/7 assay performed 72 h after 50 nM doxorubicin treatment. (**f**) Detection of doxorubicin-positive cells 72 h after 50 nM doxorubicin treatment of A375 and A375 AC-null cells. (**g**) Statistical analysis of doxorubicin-positive cell assay performed 72 h after 50 nM doxorubicin treatment. (**h**) Detection of Annexin V-positive cells 24 h after 50 nM doxorubicin treatment of A375 and A375 AC-null cells. (**i**) Statistical analysis of Annexin V-positive cell assay performed 24 h after 50 nM doxorubicin treatment. The statistical analyses for Caspase 3/7 and Annexin V were performed using Student's *t* test (*p < 0.05, **p < 0.01, ***p < 0.001). Data are expressed as mean ± SD. Experiments were performed in three independent experiments with three technical replicates each.

Wild-type (WT) and A375 AC-null cells were treated for 48 h with ascending concentrations (50–200 nM) of doxorubicin. Cell viability assay showed that WT cells were significantly more resistant to doxorubicin treatment than AC-null cells (Fig. 1a). Moreover, flow cytometry quantification of caspase 3/7 positive cells showed that AC-null cells exhibited a larger number of apoptotic events (caspase 3/7+) after 48 h and 72 h incubation with doxorubicin (50 nM) (Fig. 1b–e). At 48 h, AC-null cells displayed increased apoptosis (30.3%) compared to WT controls (12.3%) (Fig. 1d,e). A similar effect was observed at 72 h, with as many as 48.2% of AC-null cells tested positive to apoptosis, compared to 16.1% in WT controls (Fig. 1b lower panel, e). The analyses also revealed that, 72 h after doxorubicin treatment, the percentage of non-apoptotic cells containing the drug was significantly higher in WT A375 cells (15.3%) than in AC-null cells (1.4%) (Fig. 1f,g). This difference might result from the emergence of doxorubicin detoxification processes or from alterations in cell division. Finally, we used the Annexin V assay to evaluate induction of early apoptosis after doxorubicin (50 nM—24 h) exposure. Figure 1 h,i shows that 42.1% of AC-null A375 cells tested positive for annexin V compared to 29.3% of WT cells. The results support previous findings indicating that AC ablation sensitizes A375 cells to chemotherapeutic agents by, among other pathways, enhancing apoptosis^5^.

### AC-null cells show no long-term recovery after doxorubicin treatment

**Figure 2:**
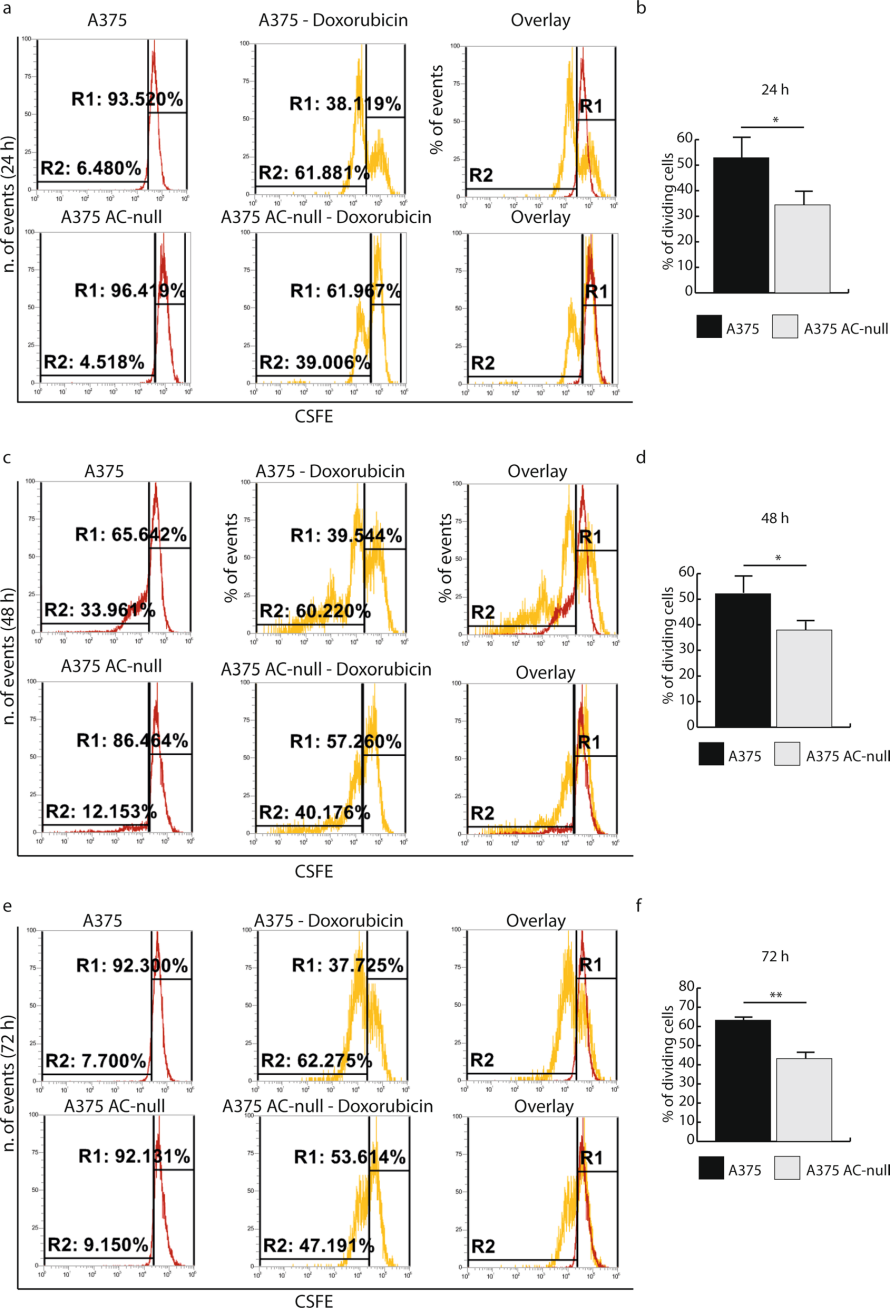
Proliferation recovery assay on A375 and A375 AC-null cells after doxorubicin administration. (**a**) CFSE decay assay performed 24 h after 50 nM doxorubicin treatment. (**b**) Statistical analysis of CFSE assay performed 24 h after 50 nM doxorubicin treatment. (**c**) CFSE decay assay performed 48 h after 50 nM doxorubicin treatment. (**d**) Statistical analysis of CFSE assay performed 48 h after 50 nM doxorubicin treatment. (**e**) CFSE decay assay performed 72 h after 50 nM doxorubicin treatment. (**f**) Statistical analysis of CFSE assay performed 72 h after 50 nM doxorubicin treatment. The CSFE statistical analyses were performed using Student's *t* test (*p < 0.05, **p < 0.01, ***p < 0.001). Data are expressed as mean ± SD. Experiments were performed in three independent experiments with three technical replicates each.

**Figure 3:**
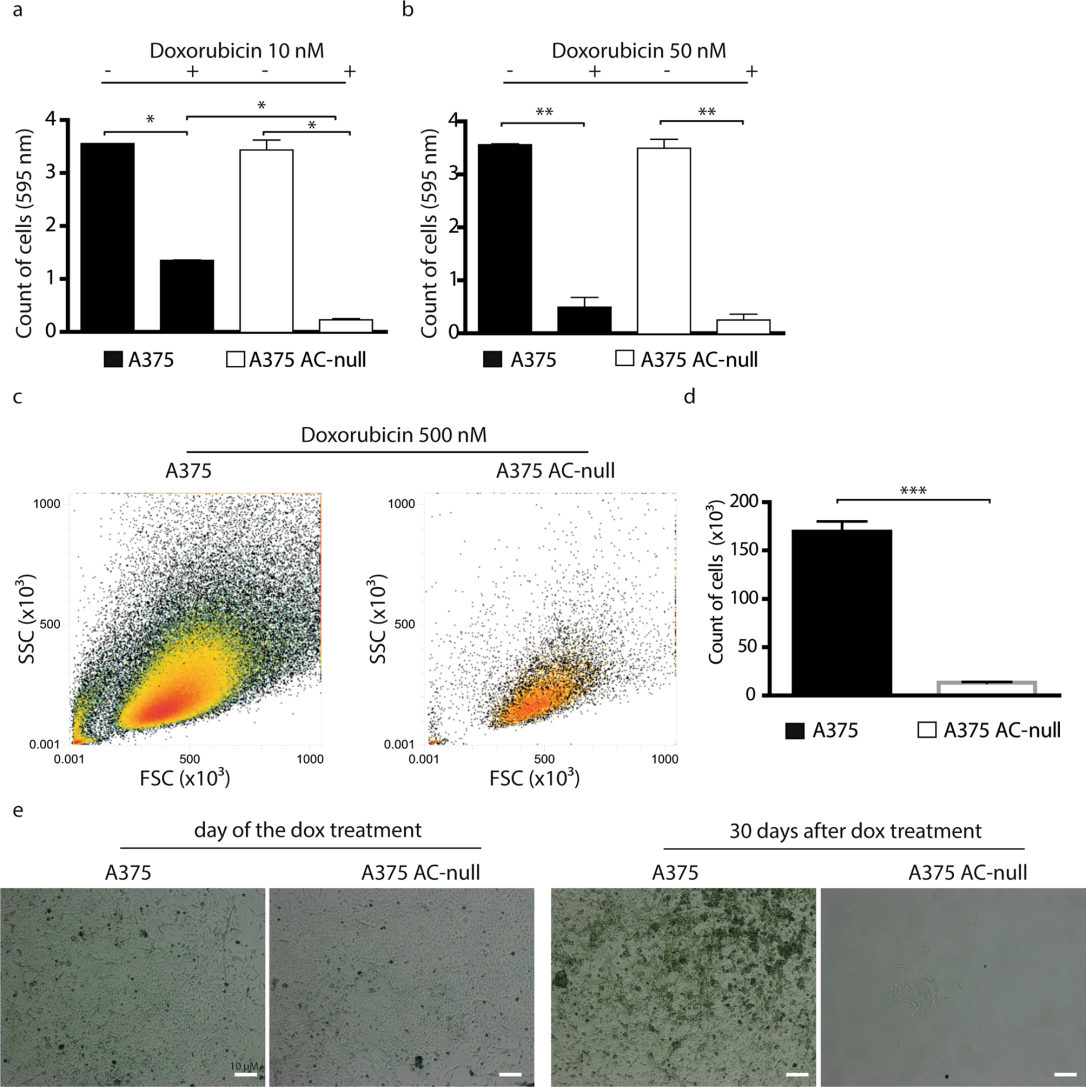
Long-term recovery assay after doxorubicin treatment of A375 and A375 AC-null cells. (**a**) crystal violet colorimetric detection of total cells 8 days after 10 nM doxorubicin administration, data are expressed as mean ± SD, the statistical analysis was performed using Student's *t* test (*p < 0.05, **p < 0.01, ***p < 0.001). (**b**) crystal violet colorimetric detection of total cells 8 days after 50 nM doxorubicin administration, data are expressed as mean ± SD. (**c**) Total number of A375 and A375 AC-null cells detection 30 days after 500 nM doxorubicin treatment using flow cytometry assay. (**d**) Statistical analysis of cells detected in (**c**), data are expressed as mean ± SD, the statistical analysis was performed using Student's *t* test (*p < 0.05, **p < 0.01, ***p < 0.001). (**e**) A375 and A375 AC-null cells images taken by optical microscope before and after the doxorubicin treatment described in (**c**).

Next, we evaluated the proliferative recovery of WT and AC-null A375 cells after doxorubicin exposure. Flow cytometry analyses revealed that, after 24 h incubation with doxorubicin, only 39% of AC-null cells completed the cell cycle compared to 61.9% of WT cells (Fig. 2a,b). Under the same conditions, the replication rates of AC-null cells were 40.2% and 47.2% after 48 h and 72 h of treatment respectively, compared to 60.2% and 62.3% for WT controls (Fig. 2c–f). This result suggests that a higher percentage of WT cells undergo cell division after doxorubicin treatment, compared to cells lacking AC. This result prompted us to monitor the cellular long-term recovery following exposure to the chemotherapeutic agent. First, we measured the recovery of WT and AC-null cells 8 days after doxorubicin incubation (24 h at 10 nM or 50 nM). Crystal violet staining showed that doxorubicin (10 nM) decreased the number of adherent cells by 93.6% in AC-null cells, compared to 62.0% in WT controls (Fig. 3a,b). No such difference was noted at 50 nM doxorubicin (Fig. 3b). We then asked whether a higher concentration of doxorubicin (500 nM) would affect long-term recovery in WT and AC-null cells. To probe so, we treated A375 and A375 AC-null cells with 500 nM doxorubicin for 24 h, recovery rate was measured 30 days after. Flow cytometry analyses revealed that WT cells exhibited a substantially stronger recovery than AC-null cells (Fig. 3c,d). Images taken by optical microscopy confirmed the increased susceptibility of A375 AC-null cells when exposed to doxorubicin, compared to their WT counterparts (Fig. 3e).

### A375 AC-null cells show impaired autophagic flux

**Figure 4:**
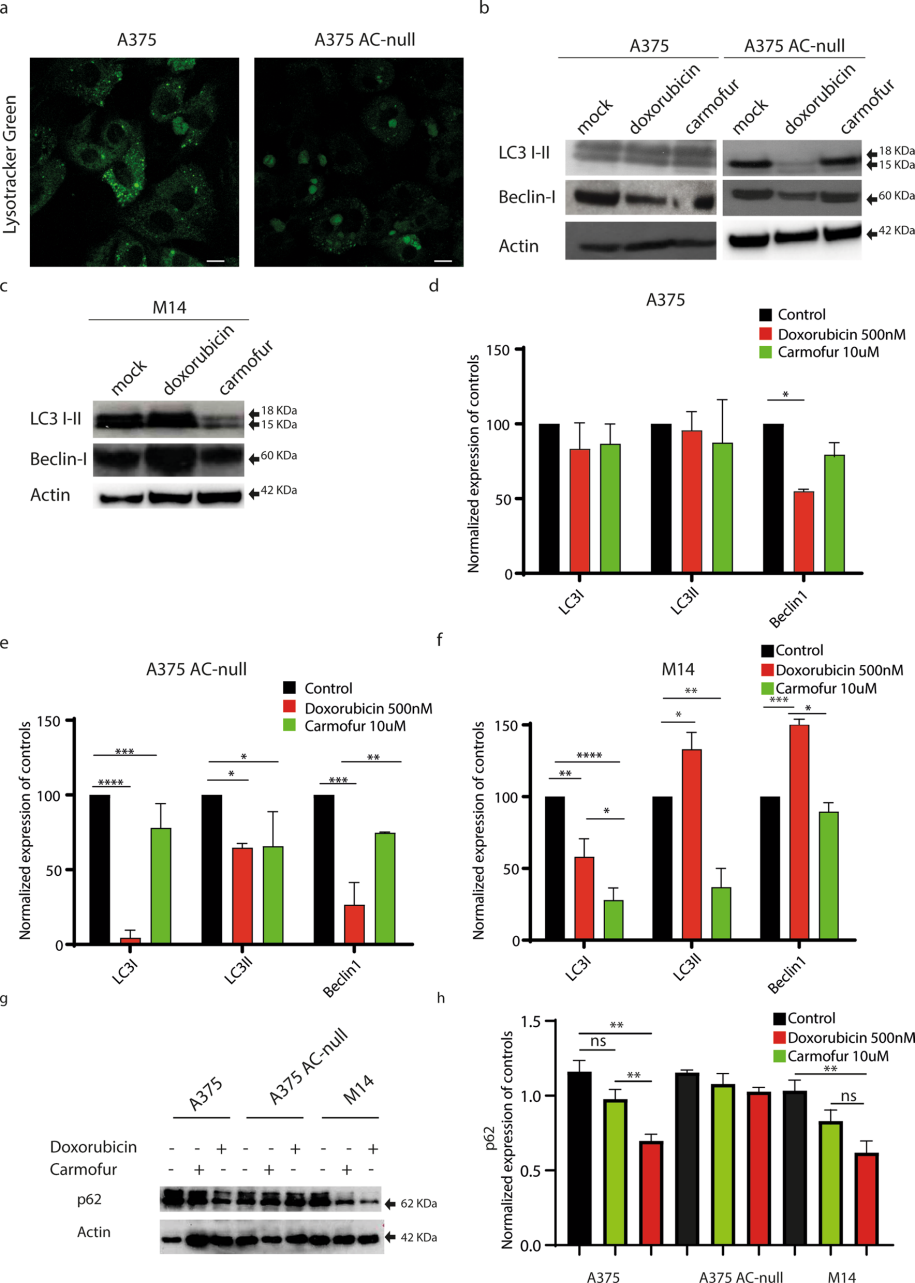
A375 AC-null cells have impaired autophagic flux. (**a**) Confocal live imaging on A375 and A375 AC-null performed using the Lysotracker Green dye shows an increased accumulation of large acidic vesicles in A375 AC-null cells. (**b**) Western blot analyses of LC3 I-II and Beclin-I expression performed on A375 and A375 AC-null cells, treated with doxorubicin (500 nM 24 h) or the AC inhibitor carmofur (10 µM 24 h) uncropped western blots are shown in Supplementary Information. (**c**) Western blot analyses of LC3 I-II and Beclin-1 expression performed on M14 melanoma cells treated with doxorubicin (500 nM) or the AC inhibitor carmofur (10 µM 24 h). (**d**) Statistical analysis of Western Blot experiments on A375 illustrated in (**b**). (**e**) Statistical analysis of Western Blot experiments on A375 AC-null illustrated in (**b**). (**f**) Statistical analysis of Western Blot experiments on M14 illustrated in (**c**). (**g**) Western blot analyses of p62 expression performed on A375, A375 AC-null and M14 cells, treated with doxorubicin (500 nM) or the AC inhibitor carmofur (10 µM) for 3 h. Chloroquine (CHQ) was added 1 h prior protein extraction at 10 µM, following autophagy evaluation guidelines^40^. (**h**) Statistical analysis of Western Blot experiments on A375 illustrated in (**g**). Western Blot statistical analyses were performed using Two-way ANOVA test (*p < 0.05, **p < 0.01, ***p < 0.001). data are expressed as mean ± SD.

Chemotherapy-induced autophagy limits the effectiveness of anticancer treatments, rendering cancer cells more resistant to induced damage^31–33^. Furthermore, there is a well-established positive correlation between AC expression and autophagy^34–36^. To evaluate the autophagic flux in WT and AC-null A375 cells, we measured the levels of expression of various autophagic proteins after doxorubicin treatment. As shown in Fig. 4a, confocal microscopy studies performed on WT and AC-null cells stained with lysotracker green showed an aberrant accumulation of large acidic vesicles in AC-null A375 cells, indicating a possible defect in cargo digestion. Consistent with this conclusion, Western blot analyses performed after doxorubicin exposure showed a lower expression of LC3 I in AC-null A375 cells compared to WT controls (Fig. 4b,d,e). Interestingly, under baseline conditions LC3 II is not detected in AC-null cells, whereas is readily detectable in WT controls. This finding suggests a defect of autophagy in A375 cells lacking AC.

Interestingly, doxorubicin incubation decreases Beclin-1 protein levels in both WT and AC-null A375 cells. Moreover, we extended our analysis to M14 melanoma cells that, among other differences with A375, exhibit high levels of AC expression^4^. As shown in Fig. 4c–f, M14 cells increase levels of LC3-II and Beclin-1 when treated with doxorubicin. Interestingly, M14 cells treated with the 5-fluorouridin prodrug Carmofur, one of the most potent AC inhibitor^38^, decrease the expression of LC3 I-II and Beclin-1 compared to controls.

Moreover, we analyzed the content of p62 protein, which levels are modulated by autophagy since it acts as a substrate during autophagic degradation^39^. We assessed p62 content 3 h after doxorubicin or carmofur treatment as suggested by Klionsky et al^40^. As shown in Fig. 4g,h, p62 decreases in M14 cells after Doxorubicin exposure, while Carmofur administration acts oppositely. As expected, p62 decreases in A375 cells treated with doxorubicin, and accumulates once A375 cells are exposed to carmofur.

**Figure 5:**
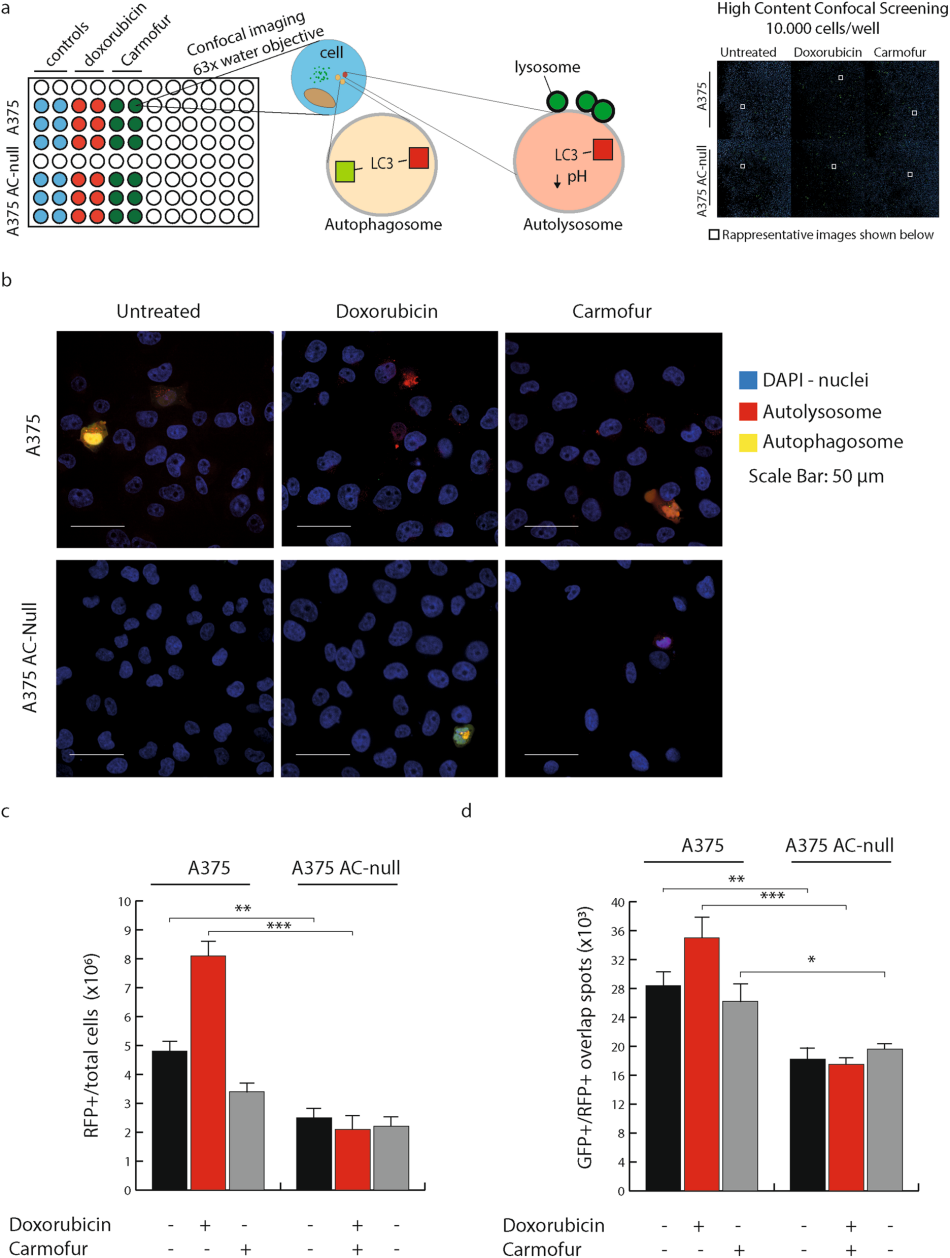
High-content confocal microscopy autophagy analysis. (**a**) Left panel illustrates the principle of high content confocal microscopy analysis. Briefly, 1 × 10^4^ pCMV-RFPLC3GFP transfected cells were treated with doxorubicin (500 nM—24 h) and Carmofur (10 µM—24 h). After treatments, cells were fixed and analyzed for GFP+ RFP+ overlapping puncta and for GFP−/RFP+ vesicle content. Around 10^3^ transfected cells/well were analyzed using Harmony algorithms, where RFP+/GFP+ vesicles are counted as autophagosomes and RFP+/GFP− vesicles are counted as autolysosomes. The outcome of this test is the following: an autophagy inducer will increase RFP+/GFP+ and RFP+/GFP− vesicles, an autophagy blocker will increase RFP+/GFP+ but decrease RFP+/GFP− vesicles, whereas an autophagy inhibitor will decrease RFP+/GFP+ and RFP+/GFP− vesicles. Right panel shows an overview of a single High-Content acquisition, in which every big square comprises hundreds of 3% overlapping images taken at × 63 magnification. (**b**) Images taken from the acquisition shown in (**a**). (**c**,**d**) Statistical analysis of RFP+/GFP+ and RFP+/GFP− vesicles revealed that A375 cells increases the RFP+/GFP+ and RFP+/GFP− vesicles when exposed to doxorubicin, compared to A375 AC-null cells, in which autophagy inhibition was detected. Statistical analyses were performed using one-way ANOVA test (*p < 0.05, **p < 0.01, ***p < 0.001). Data are expressed as mean ± SD.

To better estimate the type of autophagic impairment caused by the lack of AC, we transfected A375 AC-null cells and WT cells with pCMV RFP-LC3-GFP plasmid. As illustrated in Fig. 5a, once RFP-LC3-GFP localizes into autolysosomes, the fluorescence of GFP is quenched due to its low pH whereas that of RFP is stable. Autophagosome formation causes an increase in the number of GFP-positive/RFP-positive puncta^37^. Once autophagosomes fuse with lysosomes, puncta become GFP-negative/RFP-positive. Autophagy induction results in the increase in both yellow and red puncta, while inhibition of autophagy decrease both yellow and red puncta^41^.

Transfected cells were analyzed by high content confocal microscopy screening. The analysis revealed that A375 cells accumulate more RFP + puncta (autolysosomes) and GFP + /RFP + puncta (autophagosomes) after doxorubicin exposure. This pattern indicates that A375 induce autophagy in response to doxorubicin, while A375 AC-null cells did not show any variation of yellow/red puncta among treatments (Fig. 5b–d).

### AC-null cells display a pro-apoptotic lipidic profile

**Figure 6:**
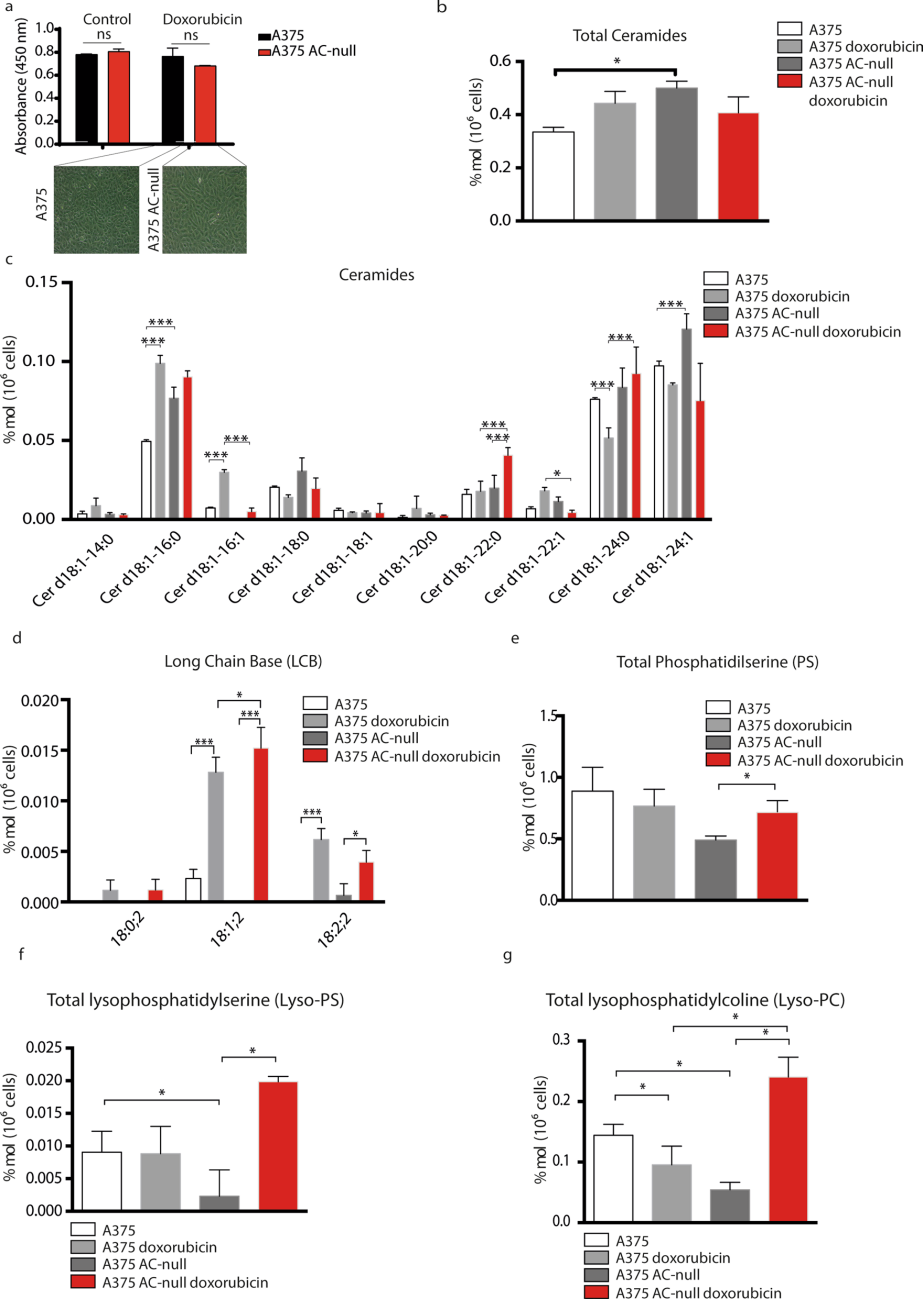
A375 AC-null cells shows an apoptotic lipidic profile. (**a**) Cell viability assay (WST-8) performed 24 h after doxorubicin treatment (500 nM). No differences were observed among groups. Statistical analysis was performed using Student’s *t* test (*p < 0.05, **p < 0.01, ***p < 0.001). data are expressed as mean ± SD. (**b**) A375 AC-null cells increase the total amount of ceramides compared to A375 WT cells. (**c**) A375 AC-null cells increase the amount of both long chain (Cer d18:1–16:0, Cer d18:1–16:1) and very-long chain ceramides (Cer d18:1–24:0) after doxorubicin exposure, compared to A375 WT cells, which increase the long chain ceramides only (Cer d18:1–16:0, Cer d18:1–16:1). (**d**) A375 AC-null cells accumulate more LCB 18:1;2 after the doxorubicin treatment compared to control A375 cells. (**e**) A375 AC-null cells accumulate more phosphatidylserine after the doxorubicin treatment compared to control A375 cells. (**f**) A375 AC-null cells have less lysophosphatidylserine than control A375 cells. The doxorubicin treatment causes an increase of lysophosphatidylserine levels only in A375 AC-null cells. (**g**) Doxorubicin treatment decrease the levels of lysophosphatidylcholine in A375 AC-null cells, while the same treatment has the opposite effect on A375 AC-null cells. The statistical analyses were performed using one-way ANOVA test (*p < 0.05, **p < 0.01, ***p < 0.001). Data are expressed as mean ± SD.

We also investigated the effects of doxorubicin on sphingolipid levels in WT and AC-null A375 cells. Cells were incubated with doxorubicin (500 nM) for 24 h and lipid extracts were analyzed by liquid-chromatography/mass spectrometry. First, we measured the cell viability of A375 AC-null cells and controls 24 h after 500 nM doxorubicin treatment. As shown in Fig. 6a, at this time-point doxorubicin did not affect cell viability among groups. The analyses revealed unstimulated AC-null cells contained a higher total amount of ceramides compared to WT controls (Fig. 6b). Of note, under these baseline conditions, AC-null cells preferentially accumulated the AC substrate Cer d18:1–16:0 ceramide^5, 42^, rather than Cer d18:1–14:0, Cer d18:1–18:0, Cer d18:1–18:1 and Cer d18:1–20:0 species (Fig. 6c). After doxorubicin exposure, Cer d18:1–16:0 levels increased in WT cells, reaching levels similar to those found in AC-null cells (Fig. 6c). Consistent with other reports^43, 44^, we also found that AC-null cells contain higher levels of very long chain ceramides such as Cer d18:1–22:0, Cer d18:1–22:1, Cer d18:1–24:0 and Cer d18:1–24:1 (Fig. 6c). The elevated levels of both long and very long ceramides are thought to balance the pro-apoptotic signal induced by accumulation of long chain ceramides only^45^. Doxorubicin exposure also increased sphingosine content in both WT and AC-null cells (Fig. 6d). This finding is consistent with previous works and may correlate with mitochondrial-dependent apoptosis^46, 47^. The levels of phosphatidylserine, lysophosphatidylserine and lysophosphatidylcholine, phospholipid species that are involved in cell death^48^ were substantially higher in AC-null cells treated with doxorubicin than in untreated AC-null cells or WT controls (Fig. 6e–g).

### A375 AC-null cells have Atg5-cleaved that switches autophagy to apoptosis

**Figure 7:**
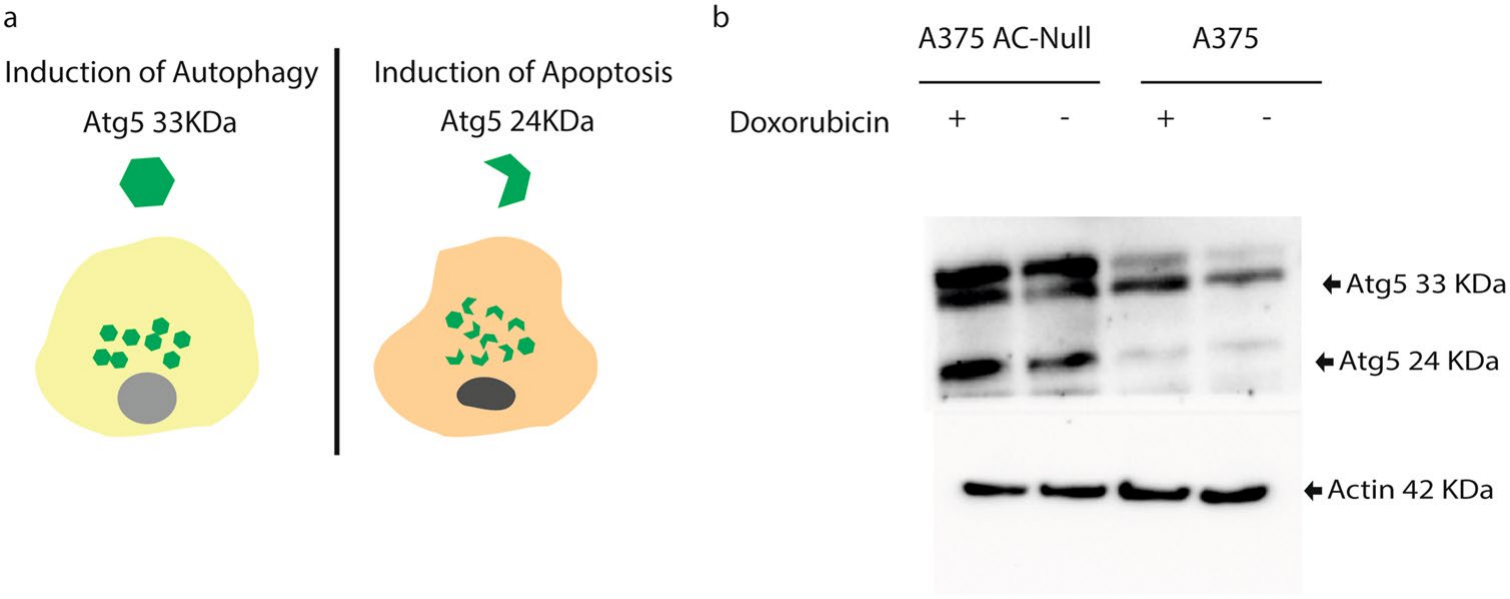
A375 AC-null cells scored positive for the pro-apoptotic Atg5 cleaved protein. (**a**) Schematic illustration of the role of Atg5 in the regulation of autophagy and apoptosis. (**b**) Western blot was performed on A375 WT and A375 AC-null cells treated with 500 nM of doxorubicin for 24 h. Western blot analysis show the presence of the pro-apoptotic cleaved Atg5 protein in the A375 AC-null cells lysate while it is not detected in the A375 WT cells. Uncropped western blots are shown in Supplementary Information.

Starting from the finding that AC-null A375 cells exhibit impaired autophagy and a pro-apoptotic lipid profile, we asked whether a link between autophagy and apoptosis might emerge in these cells after doxorubicin treatment. To address this question, we analyzed the integrity of the Atg5 protein, which is involved in both autophagy and apoptosis^49^. As illustrated in Fig. 7a, full-length 33 kDa Atg5 is required for autophagosome formation^50^, while the truncated 24 kDa form activates apoptosis^49, 51^. Western blot analyses showed that, both under baseline conditions and after doxorubicin incubation, AC-null A375 cells contained significant levels of the truncated form of Atg5, which was not detectable in WT controls (Fig. 7b).

## Discussion

The present work investigated the role of the lysosomal cysteine amidase AC, in the response of A375 melanoma cells to the anthracycline chemotherapeutic doxorubicin. As expected from previous studies^5^, AC deletion caused a marked enhancement of doxorubicin-induced apoptosis. Furthermore, those AC-null cells that resisted apoptosis failed to restore cell replication up to 30 days after exposure to the drug. To elucidate the mechanism underlying this long-lasting cell-cycle impairment, we probed the autophagic flux in wild-type and AC-null A375 cells. We found that cells lacking the enzyme exhibit an aberrant accumulation of autophagosomes which is reminiscent of the cytopathological events occurring in Farber disease^52^. Similar results were obtained in AC-overexpressing M14 melanoma cell treated with doxorubicin and the potent AC inhibitor carmofur. Experiments aimed at exploring possible links between autophagy and apoptosis, revealed that AC-null cells contained detectable levels of the calpain-cleaved 24-kDa Atg5 protein, which hinders autophagy to activate apoptosis^49, 51^.

The inability of AC-null cells to restore the cell cycle following doxorubicin treatment prompted us to hypothesize that AC ablation may create an aberrant ceramide accumulation in lysosomes, which might result in impaired autophagy. The mechanism underpinning this dysregulation is unknown, but three hypotheses can be formulated. First, since AC is the only enzyme that digests long-chain ceramides in lysosomes, it is possible that the accumulation of these lipid substances might overburden the lysosome and mechanically disable cargo digestion. Second, ceramides are presented to AC by the protein saposin-D. Saposine-defective mice exhibit altered autophagy and an abnormally high number of unprocessed autophagosomes^53^. It is possible that ceramide accumulation in AC-null cells sequesters saposin-D, causing a phenotype similar to the one described in saposin-deficient mice. Third, Liu and collaborators^54^ have shown that lysosomal long-chain ceramides activate cathepsin-D, a lysosomal protease that suppresses autophagy. It is thus possible that the marked reduction in autophagy observed in AC-null cells might be caused by ceramide-induced cathepsin-D activation. Further experimentation is needed to test these hypotheses.

The findings show that AC deletion causes profound alterations in the lipid profile of A375 cells following incubation with doxorubicin. Consistent with previous studies on mammary tumours, we found that doxorubicin treatment increased total ceramide content in wild-type A375 cells^45^. Moreover, as previously described^5^, untreated AC-null cells accumulated larger amounts of ceramides than did wild-type controls. A more granular inspection of the lipidomic data revealed that exposure to doxorubicin decreased the levels of ceramide d18:1–22:0 and d18:1–24:0 in wild-type cells, whereas it exerted an opposite effect in AC-null cells. The role of very long-chain ceramides in the regulation of apoptosis is still unclear. There are evidences that long-chain ceramides may activate apoptosis whereas very long ceramides may not^55^. Other studies suggest that different ceramide species may be involved in distinct phases of the apoptotic process: apoptotic cells may initially produce ceramide d18:1–16:0 and d18:1–18:0 followed by formation of ceramide d18:1–24:0 when the process reaches its final stages^56^. Our data show that AC-null cells accumulate ceramide d18:1–22:0 and d18:1–24:0, and are more prone to activate apoptosis, whereas chemotherapy-resistant wild-type cells reduce their intracellular amounts of ceramide d18:1–22:0 and d18:1–24:0 after incubation with doxorubicin.

It is described that doxorubicin enhances ceramide production mainly through de novo synthesis pathway, which results in a prolonged ceramide elevation and plays a main role in ceramide-mediated apoptotic signalling^57^. Probably AC plays a role in doxorubicin resistance by limiting ceramide accumulation derived from ceramide synthases. The lack of AC might generate an amplification loop that increases the apoptotic induction. Of note, our lipidomic analyses show that doxorubicin may alter the intracellular content of several other lipid classes, in addition to ceramides. Whereas the drug increased the amount of LCB in both wild-type and AC-null cells, it exerted a different effect on lyso-PS and lyso-PC, the levels of which were increase in AC-null cells after doxorubicin treatment. The presence of lyso-PS on the surface of apoptotic cells enhances efferocytosis by macrophages^58^. Lyso-PC, another lysophospholipid that we found to accumulate preferentially in AC-null cells after doxorubicin incubation is known to stimulate apoptosis in various cell types, including endothelial^59^ and Huh-7 hepatoma cells^60^. Together, our lipidomic analyses suggest that doxorubicin creates a pro-apoptotic lipid signature in AC-null cells, which is not limited to the increase of long-chain ceramides, but also involves the very-long chain ceramide species d18:1–22:0 and d18:1–24:0 as well as other pro-apoptotic phospholipid mediators.

Finally, our results suggest that wild-type and AC-null A375 cells follow two separate paths after doxorubicin exposure. Wild-type cells turn on autophagy and fully recover from doxorubicin-induced damage. By contrast, AC-null cells fail both to activate autophagy and recover. This dichotomy is paralleled by a marked difference in the integrity of Atg5, a 33-kDa protein that is required for autophagosome formation and can switch autophagy to apoptosis when cleaved into a 24 kDa protein by calpain^49^. We found that the truncated form of Atg5 is readily detectable in AC-null A375 cells, but not in wild-type control cells. A similar connection between autophagy, apoptosis and AC expression was recently observed when AC-null cells are exposed to nutrient deprivation^61^.

In conclusion, the present work highlights changes that affect melanoma cells during incubation with doxorubicin in A375 melanoma cells lacking AC. We found that the remarkable reduction in recovery rate after doxorubicin treatment is strictly bond with the impairment of autophagy, that force the AC-inhibited cells into apoptotic path.

## Experimental procedures

### Cell cultures

Human epithelial melanoma A375 cells were purchased from American Type Culture Collection (Manassas, VA) and A375 AC-null cells were obtained by CRISPR-Cas9 gene-editing, as described^5^. Cells were cultured in Dulbecco’s Modified Eagle’s Medium (DMEM) supplemented with 10% fetal bovine serum (FBS, 2 mM l-glutamine and antibiotics (penicillin, streptomycin) at 37 °C and 5% CO_2_.

### Doxorubicin treatments

Doxorubicin (Merk, D2975000) was used in a range from 0.1 to 0.8 µg/mL, based on average doxorubicin plasma concentration measured 1–10 h post-infusion in human patients^60^.

### Flow cytometry: long-term recovery, apoptosis, and cell cycle assay, annexin-V assay

Cells (10^5^/well) were seeded 24 h before treatment with doxorubicin (500 nM). After 24 h, the cells were rinsed in phosphate-buffered saline (PBS) at pH 7.4. Cells were grown for 20 days in DMEM at 37 °C in a humidified 5% CO_2_ atmosphere. They were rinsed with PBS and the medium was replaced with DMEM for 10 days before flow cytometry, which was performed using the Attune NxT Acoustic Focusing Cytometer (Invitrogen, Thermo Fisher Scientific).

Apoptosis was measured using the CellEventTM Caspase-3/7 Green Flow Cytometry Assay Kit (Thermo Fisher Scientific). Cells (10^5^/well) were seeded 24 h before treatment with doxorubicin (50 nM). After 24 h, 48 h or 72 h cells were harvested in 1 mL of PBS containing 2% bovine serum albumin (BSA, Sigma). Cell samples were incubated with CellEventTM (Invitrogen, Thermo Fisher Scientific) Caspase-3/7 Green Detection Reagent for 30 min at 37 °C, protected from light. Flow cytometry analysis was performed as outlined above. Apoptosis was also evaluated using the FITC Annexin V/Dead Cell Apoptosis Kit (Invitrogen, Thermo Fisher Scientific). Cells (10^5^/well) were seeded 24 h before treatment with doxorubicin (50 nM). The samples were harvested, washed with PBS and pelleted by centrifugation. Cells were resuspended in annexin-binding buffer and added the FITC annexin V. Results were analyzed by flow cytometry. Cell cycle assays were performed using CellTraceTM Cell Proliferation Kits (Invitrogen, Thermo Fisher Scientific). Cells (10^5^/well) were harvested and pelleted by centrifugation. The samples were resuspended in PBS containing CellTraceTM CFSE dye (2uM) and incubated for 20 min at room temperature, protected from light. Samples were incubated with DMEM without FBS for 5 min. Cells were pelleted and resuspended in DMEM 10% FBS. Stained cells (10^5^/well) were seeded and before the analysis treated with doxorubicin (50 nM) for 24, 48 and 72 h each. Results were analyzed by flow cytometry.

### Cell viability assay

Cell viability was assessed using the WST assay (Cell Counting Kit-8 Sigma). Cells (15 × 10^3^) were seeded in 96-well plates 24 h before treatment with various concentration of doxorubicin (50 nM, 75 nM, 100 nM, 200 nM). 48 h later, the samples were incubated with WST for 1 h at 37 °C. Same experiment was performed after 24 h of 500 nM of doxorubicin treatment. Results were analyzed using Varioskan Lux software (Thermo Fisher Scientific).

### Confocal microscopy screening

Cells (1 × 10^4^/well) were transfected with pCMV RFP-LC3-GFP plasmid using Lipofectamine LTX (Thermofisher, USA) following manufacturer’s instructions. RFP-LC3-GFP transfected cells were cultured in cell-carrier Ultra plates (Perkin-Elmer, UK) overnight. Media was then replaced with DMEM culture medium containing the indicated chemicals for the indicated time periods. Nuclei were stained using DAPI (Thermofisher, USA). Images were acquired with Operetta CLS confocal fluorescent microscope (Perkin-Elmer, UK). The analysis was performed using Harmony software (Perkin-Elmer, UK) on over 45 fields/well, taking images with 63 × objective and analyzing an average of 10^3^ transfected cells. We counted the amount of either GFP-LC3 puncta overlayed or not with RFP-LC3 in every transfected cell detected by the instrument.

### Western blot

Cells (5 × 10^5^) were rinsed with PBS and incubated with RIPA Lysis buffer (Millipore, Massachusetts, USA) containing protease inhibitors (PierceTM Protease Inhibitor, Thermo Scientific) for 10 min on ice. The cells were harvested with a cell scraper and lysed at 4 °C overnight. Samples were centrifuged at 13,000×*g* for 10 min. Supernatants were collected, and protein content was measured by the Bradford method (Bio-Rad, Hercules, USA). Proteins were separated on polyacrylamide gels (NuPAGETM 4–12% Bis–Tris Gel Invitrogen). Immunoblotting was performed using a rabbit polyclonal anti-LC3B antibody (1:1000; 700712, Invitrogen, Thermo Fisher Scientific), rabbit anti-ATG5 (1:1000; AO731, Sigma, St. Louis, USA), mouse anti-β-actin (1:1000; A1978, Sigma, St. Louis, USA), mouse anti-SQSTM1/p62 (1:1000, ab56416, Abcam, Cambridge, UK) Density of the bands was quantified with ImageJ. Some of the blots in the following articles were analyzed using the Chemidoc acquisition system, as illustrated in Supplementary Information, in which a single blot uncropped is shown.

### Quantitative shotgun lipidomics

The analysis of lipids extracts was performed on total cell lysates, following a protocol previously reported^62,63^.

### Statistical analyses

Results were analyzed using GraphPad Prism software and all numerical values were expressed as mean ± SD. For FACS and Western blot experiments, statistical significance was assessed using the Student's *t* test (*p < 0.05, **p < 0.01, ***p < 0.001). One-way ANOVA followed by Tukey’s test was used for statistical analyses of cell viability and lipidomic investigation. The homogeneity of variances was performed using Levene’s test. Differences were considered significant at *p < 0.05, **p < 0.01, ***p < 0.001, and ****p < 0.0001.

## Supplementary Information

Supplementary Information.
