# Publisher Correction: Acid ceramidase controls apoptosis and increases autophagy in human melanoma cells treated with doxorubicin

**DOI:** 10.1038/s41598-021-99797-6

**Published:** 2021-10-07

**Authors:** Michele Lai, Rachele Amato, Veronica La Rocca, Mesut Bilgin, Giulia Freer, Piergiorgio Spezia, Paola Quaranta, Daniele Piomelli, Mauro Pistello

**Affiliations:** 1 Retrovirus Centre, Department of Translational Medicine and New Technologies in Medicine and Surgery, University of Pisa, Pisa, Italy; 2 Institute of Life Science, Scuola Sant’Anna Pisa, Pisa, Italy; 3 Cell Death and Metabolism Unit, Center for Autophagy, Recycling and Disease, Danish Cancer Society Research Center, Copenhagen, Denmark; 4 Anatomy and Neurobiology, University of California, Irvine, CA USA; 5 Virology Unit, Pisa University Hospital, Pisa, Italy

Correction to: *Scientific Reports* 10.1038/s41598-021-90219-1, published online 27 May 2021

The original version of this Article contained errors.

In Figure 5 the labels at the top and bottom of the figure were incorrectly captured. The original Figure 5 and accompanying legend appear below.

**Figure 5:**
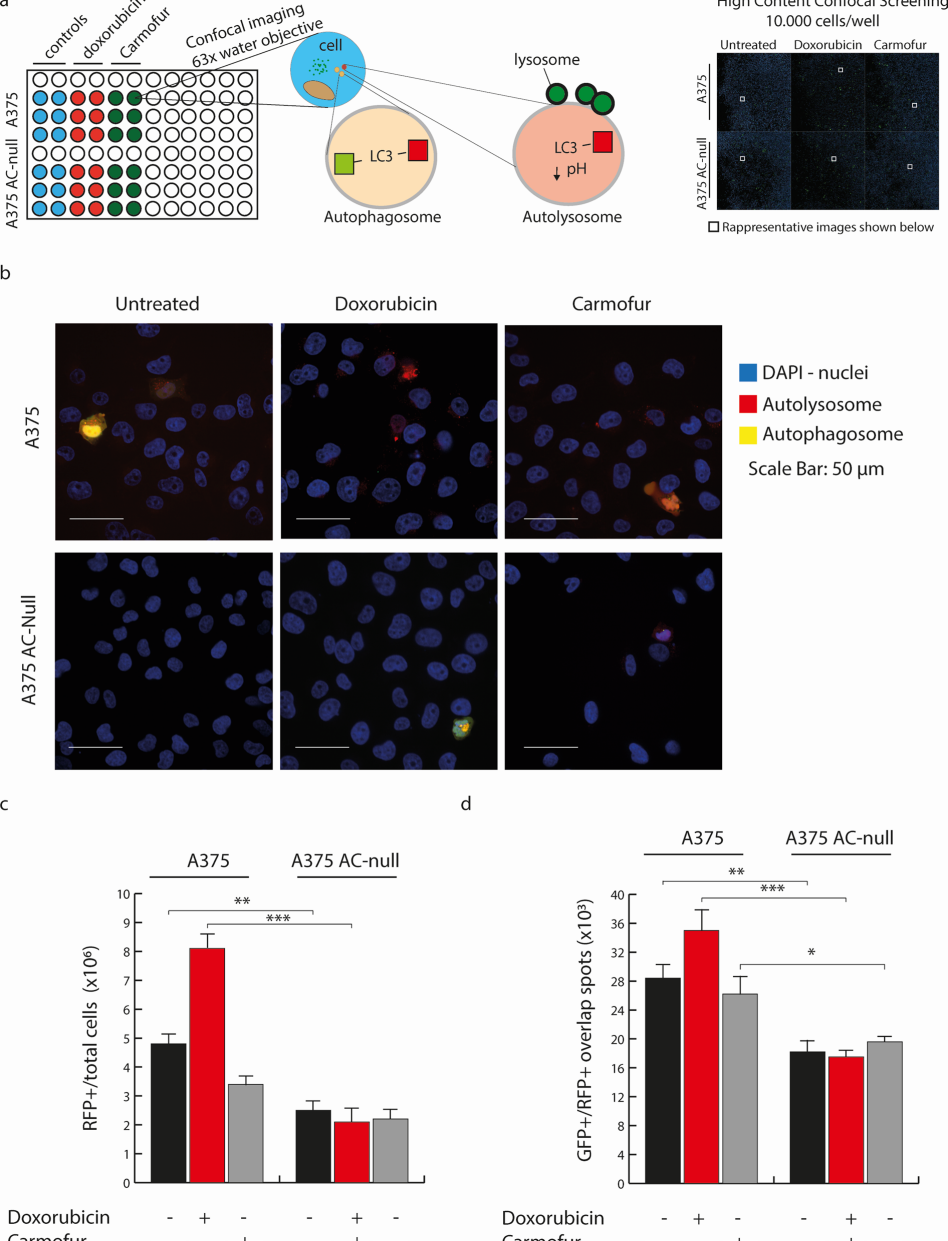
High-content confocal microscopy autophagy analysis. (**a**) Left panel illustrates the principle of high content confocal microscopy analysis. Briefly, 1 × 10^4^ pCMV-RFPLC3GFP transfected cells were treated with doxorubicin (500 nM—24 h) and Carmofur (10 µM—24 h). After treatments, cells were fixed and analyzed for GFP + RFP + overlapping puncta and for GFP − /RFP + vesicle content. Around 10^3^ transfected cells/well were analyzed using Harmony algorithms, where RFP + /GFP + vesicles are counted as autophagosomes and RFP + /GFP − vesicles are counted as autolysosomes. The outcome of this test is the following: an autophagy inducer will increase RFP + /GFP + and RFP + /GFP − vesicles, an autophagy blocker will increase RFP + /GFP + but decrease RFP + /GFP − vesicles, whereas an autophagy inhibitor will decrease RFP + /GFP + and RFP + /GFP − vesicles. Right panel shows an overview of a single High-Content acquisition, in which every big square comprises hundreds of 3% overlapping images taken at × 63 magnification. (**b**) Images taken from the acquisition shown in (**a**). (**c**,**d**) Statistical analysis of RFP + /GFP + and RFP + /GFP − vesicles revealed that A375 cells increases the RFP + /GFP + and RFP + /GFP − vesicles when exposed to doxorubicin, compared to A375 AC-null cells, in which autophagy inhibition was detected. Statistical analyses were performed using one-way ANOVA test (*p < 0.05, **p < 0.01, ***p < 0.001). Data are expressed as mean ± SD.

In addition, the Author Contributions section was incomplete.

“Conceptualization, M.L. and R.A.; methodology, V.L.R.; validation and statistical analysis, R.A; lipidomic assays M.B; revision of manuscript, G.F., D.P. and MP; writing—original draft preparation, V.L.R; writing review and editing, M.L., R.A.; Confocal screenings: M.L., P.S. All authors have read and agreed to the published version of the manuscript.”

now reads:

“Conceptualization, M.L. and R.A.; methodology, V.L.R.; validation and statistical analysis, R.A; lipidomic assays M.B; data curation, funds management PQ; revision of manuscript, G.F., D.P. and MP; writing—original draft preparation, V.L.R; writing review and editing, M.L., R.A.; Confocal screenings: ML, P.S. All authors have read and agreed to the published version of the manuscript.”

The original Article has been corrected.

