# Supplementary material for: Acid ceramidase controls apoptosis and increases autophagy in human melanoma cells treated with doxorubicin
Source: Sci Rep. 2021 May 27;11:11221. doi: 10.1038/s41598-021-90219-1 (PMC8159975; doi:10.1038/s41598-021-90219-1)
Supplement: Supplementary file 1 — Supplementary Information. [file 41598_2021_90219_MOESM1_ESM.docx]

- **Acid Ceramidase controls apoptosis and increase autophagy in human melanoma cells treated with Doxorubicin**
- **Supplementary Information 1**

Michele Lai^1^*, Rachele Amato^2^, Veronica La Rocca^2^, Mesut Bilgin^3^, Giulia Freer^1^, Piergiorgio Spezia^1^, Paola Quaranta^1^, Daniele Piomelli^4^ and Mauro Pistello^1,5^

^1^ Retrovirus Centre, Department of Translational Medicine and New Technologies in Medicine and Surgery, University of Pisa, Italy

^2^ Institute of Life Science, Scuola Sant’Anna Pisa, Italy

^3^ Cell Death and Metabolism Unit, Center for Autophagy, Recycling and Disease, Danish Cancer Society Research Center, Copenhagen, Denmark

^4^ Anatomy and Neurobiology, University of California, Irvine, California

1. Virology Unit, Pisa University Hospital, Pisa, Italy

*Corresponding author: Michele Lai [michele.lai@unipi.it](mailto:michele.lai@unipi.it)

**Supplementary Information 1** – uncropped Western Blots –

**Western blots: A375 cell line**


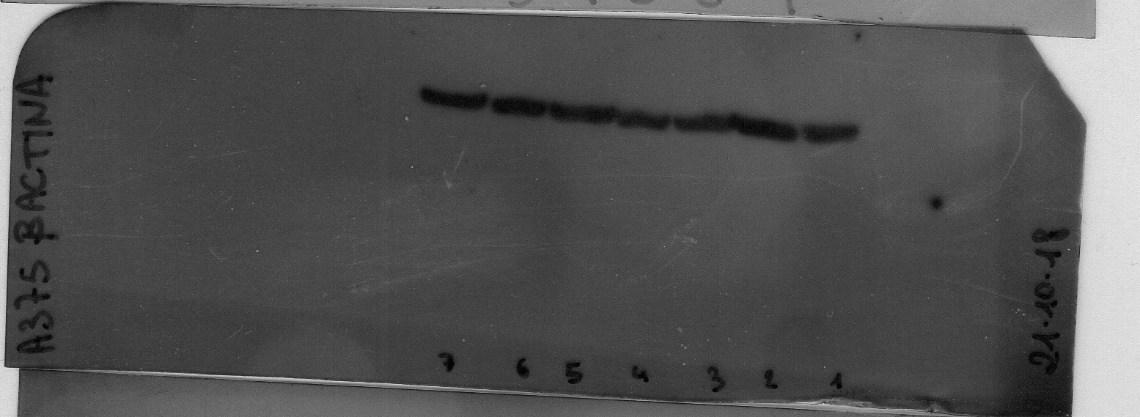
**Β-actin – A375 – Film exposure**

**Beclin 1 – A375 – Film exposure**


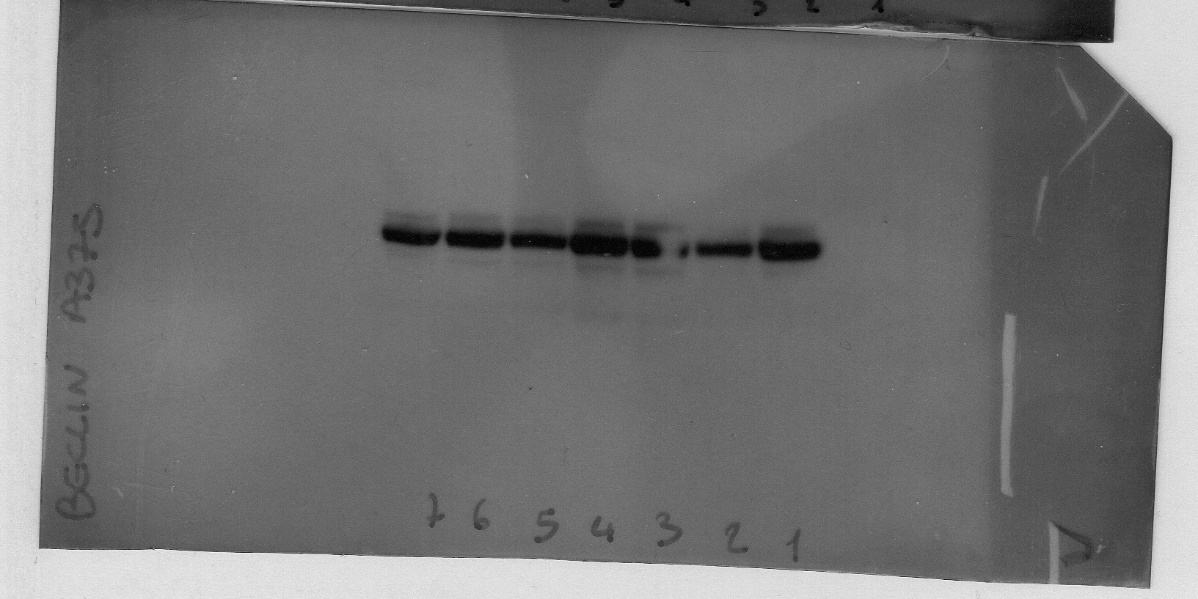


**
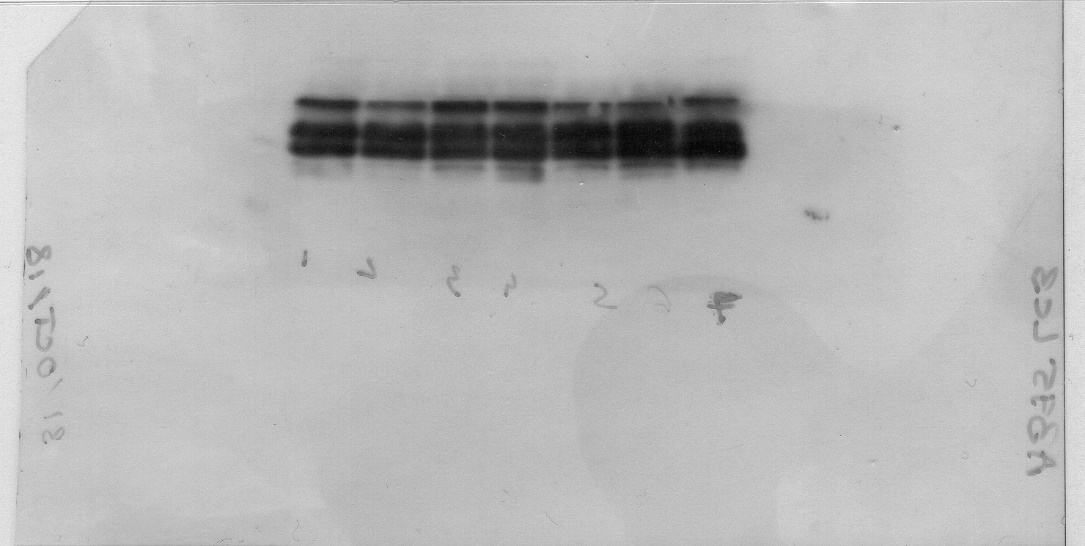
LC3-I-II A375 – Film exposure**

**ATG5- A375 – Chemidoc acquisition and analysis**

**Western Blots: A375 AC Null cells**

**β-Actin – A375 AC Null – Chemidoc acquisition and analysis**


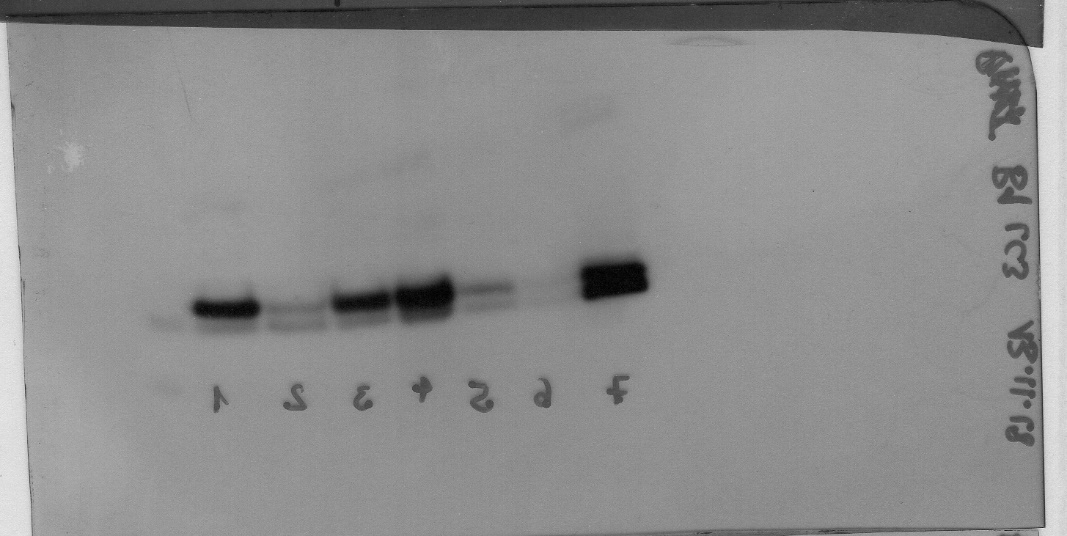
**LC3 I-II -A375 AC Null Film exposure**


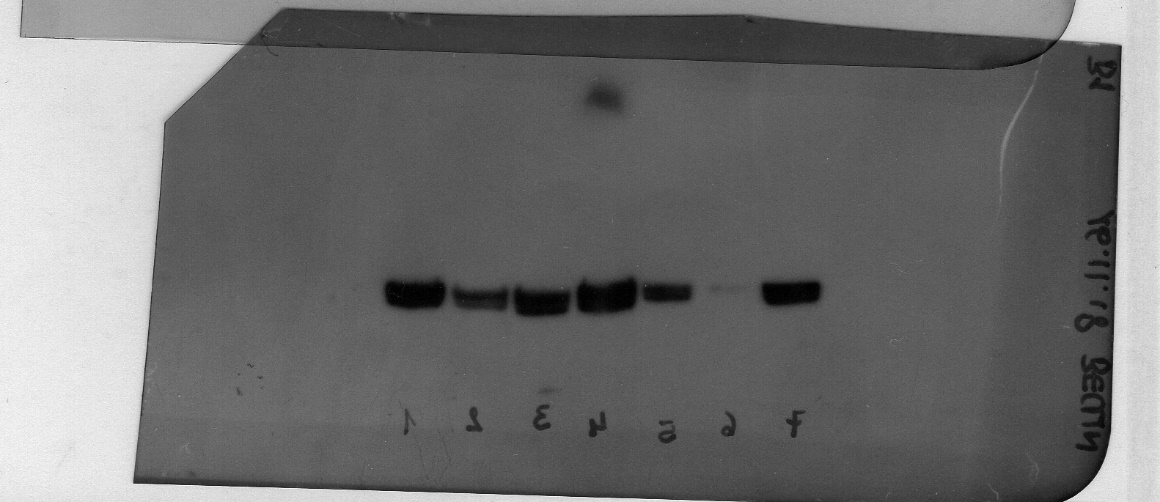
**ATG5 – A375 AC-Null Chemidoc Acquisition and Analysis**

**
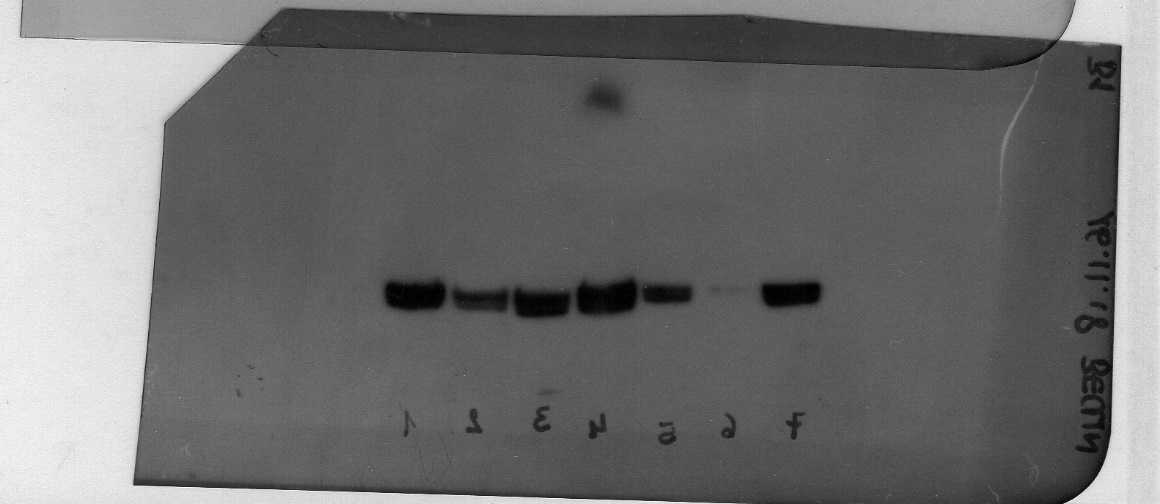
Beclin-I A375 AC-Null Film Exposure**

**WESTERN BLOTS - M14 cell line**

**β-actin Chemidoc Acquisition**

**Beclin-1 M14 cell line Film Exposure**

**
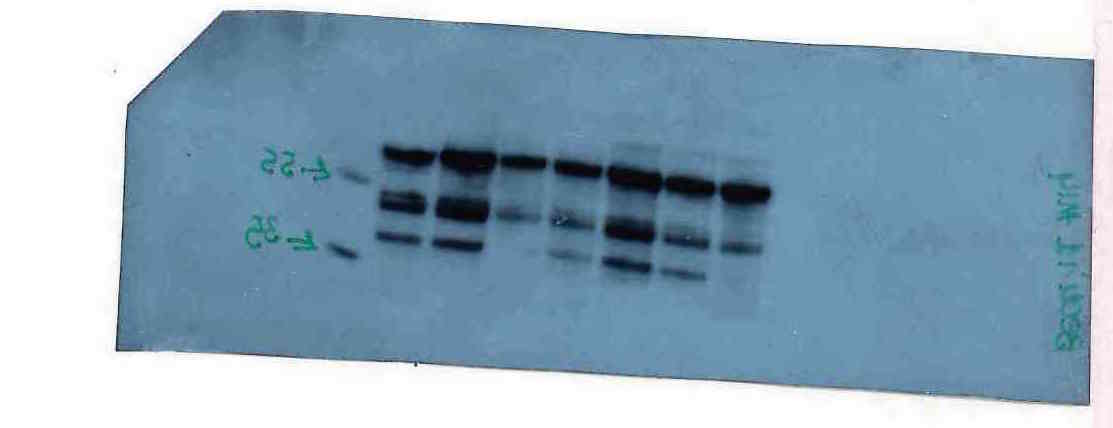
**

**LC3 I-II M14 cell line Film Exposure**

**P62 Film Exposure: ASAH null A375, M14**

**
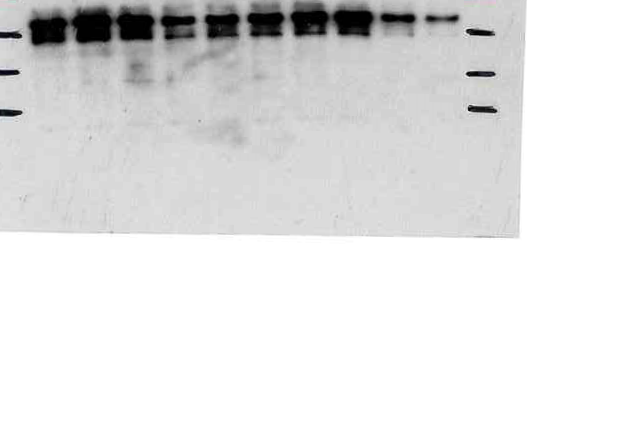
**

**Actin Film exposure ASAH null A375 M14**

**
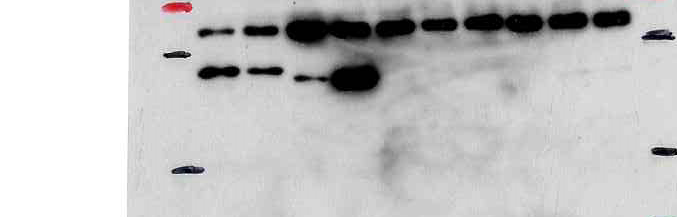
**

**Atg5 Chemidoc Acquisition**

**
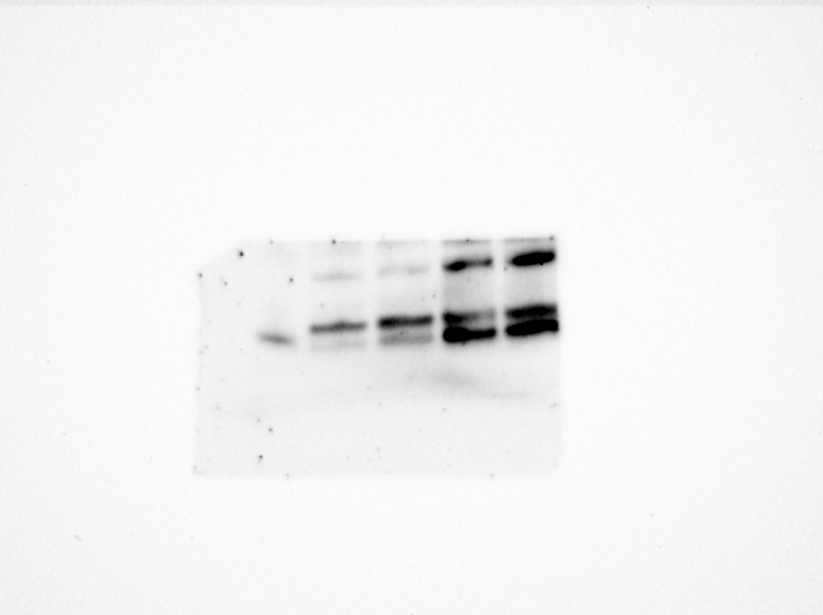
**

**β-actin Chemidoc Acquisition**

**
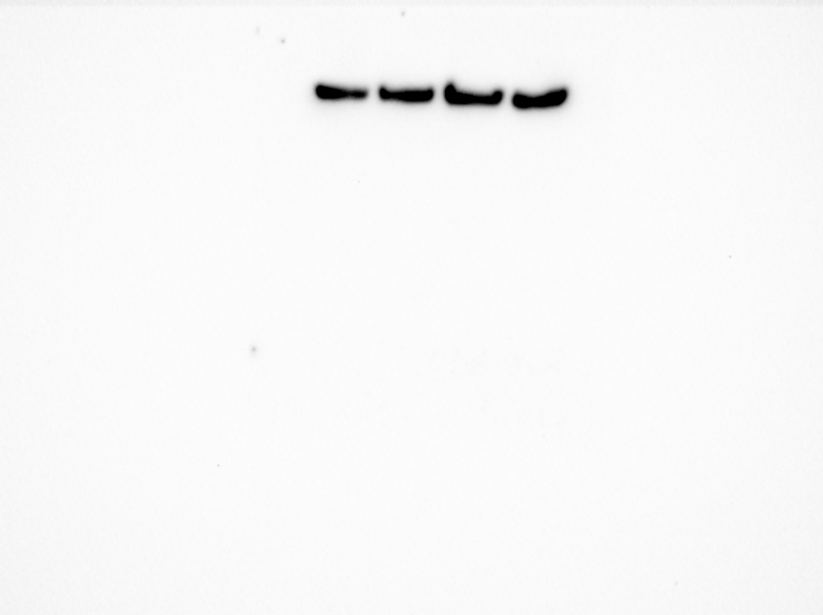
**
